# Epigenetic Activity of Peroxisome Proliferator-Activated Receptor Gamma Agonists Increases the Anticancer Effect of Histone Deacetylase Inhibitors on Multiple Myeloma Cells

**DOI:** 10.1371/journal.pone.0130339

**Published:** 2015-06-19

**Authors:** Nassera Aouali, Angeliki Broukou, Manon Bosseler, Olivier Keunen, Vincent Schlesser, Bassam Janji, Valerie Palissot, Philippe Stordeur, Guy Berchem

**Affiliations:** 1 Laboratory of Experimental Hemato-Oncology, LHCE, Luxembourg Institute of Health (LIH), Strassen, Luxembourg; 2 Laboratory Neuro-Oncology, Norlux, Luxembourg Institute of Health (LIH), Strassen, Luxembourg; 3 Laboratory of Hematology, Centre Hospitalier de Luxembourg (CHL), Strassen, Luxembourg; 4 Biotechnology Department, Experimental Infectious Diseases Platform, CER Group, Marloie, Belgium; University of Oxford, UNITED KINGDOM

## Abstract

Epigenetic modifications play a major role in the development of multiple myeloma. We have previously reported that the PPARγ agonist pioglitazone (PIO) enhances, in-vitro, the cytotoxic effect of the Histone deacetylase inhibitor (HDACi), valproic acid (VPA), on multiple myeloma cells. Here, we described the development of a new multiple myeloma mouse model using MOLP8 cells, in order to evaluate the effect of VPA/PIO combination on the progression of myeloma cells, by analyzing the proliferation of bone marrow plasma cells. We showed that VPA/PIO delays the progression of the disease and the invasion of myeloma cells in the bone marrow. Mechanistically, we demonstrated that VPA/PIO increases the cleavage of caspase 3 and PARP, and induces the acetylation of Histone 3 (H3). Furthermore, we provided evidence that PPARγ agonist is able to enhance the action of other HDACi such as Vorinostat or Mocetinostat. Using PPARγ antagonist or siPPARγ, we strongly suggest that, as described during adipogenesis, PIO behaves as an epigenetic regulator by improving the activity of HDACi. This study highlights the therapeutic benefit of PIO/VPA combination, compared to VPA treatment as a single-arm therapy on multiple myeloma and further highlights that such combination may constitute a new promising treatment strategy which should be supported by clinical trials.

## Introduction

Multiple myeloma (MM) is a hematological disorder of the plasma cell population in bone marrow, characterized by increased proliferation of neoplastic plasma cells, overproducing a monoclonal immunoglobulin (M component) detected in blood or urine [1]. Common clinical symptoms include lytic bone lesions, renal failure, hypercalcemia and fatigue due to severe anemia.

Despite the emergence of new molecules for multiple myeloma, the disease is still incurable. Indeed, the major obstacle to chemotherapy treatment is the development of drug resistance. To bypass this, the pharmaceutical industry has developed new molecules targeting numerous molecular pathways [2–4], however, all patients relapse sooner or later. To improve the treatment of relapsed or refractory MM patients, combination of several drugs, belonging to different pharmaceutical classes, is the proposed option by clinicians [5].

It has been clear that epigenetic modifications play a major role in cancer development [6] resulting from aberrant histone acetylation. The chromatin structure is maintained by two protein families, the Histone Deacetylases (HDACs) and Histone Acetyl Transferases (HATs). The acetylation state of histones modifies the conformation of chromatin and therefore modulates genes expression [7]. Such properties of Histone Deacetylases inhibitors (HDACi) make them promising new treatment regimens in hematological and other malignancies [8, 9]. Several HDACi are currently in clinical trials [9]. In this study we used the clinically approved HDACi, valproic acid (VPA) drug used for over two decades in the treatment of epilepsy and bipolar disorders. In 1997, VPA emerged as an antineoplastic agent, when findings indicated that VPA was able to inhibit cell proliferation and induce differentiation of neuroectodermal tumor cells in-vivo [10].

The anti-tumor activity of Peroxisome Proliferator-Activated Receptor gamma (PPARγ) agonists has been reported in 1997 [11, 12]. PPARγ is a ligand-activated transcription factor belonging to the steroid/thyroid nuclear receptor family and required for differentiation and lipid metabolism [13]. Its expression is induced during adipogenesis and it plays also a key role in the establishment of the transcriptome of terminally differentiated fat cells [14]. PPARγ is activated following the interaction with their ligands, including various natural molecules like long chain polyunsaturated fatty acids [15], cyclopentone prostaglandin [16], synthetic ligands such as the anti-diabetic thiazolinedione (TZD) and non-thiazolinedione molecules [17, 18]. Numerous studies have shown that PPARγ agonists influence broad biological processes, including cancer. Thus, PPARγ agonists cause cell cycle arrest and promote apoptosis in human cancer cell lines and animal models [12, 19]. In addition, molecules belonging to the TZD family exhibit a chemotherapy effect in several leukemia cell lines as well as in solid tumors [20].

We have previously reported that the cytotoxic effect of VPA on MM cell lines in-vitro was increased when combined with PIO [21], with a stronger synergistic effect on MM cells isolated from patients’ bone marrow (BM) and blood. In this study, we carried out experiments to investigate the molecular mechanism of VPA/PIO combination on a MM mouse model, exhibiting symptoms comparable to those seen in MM patients. Furthermore, we showed that a combinatorial treatment of VPA/PIO increases the lifespan of mice in-vivo and delays the development of MM compared to VPA treatment alone. In addition, we observed that the concentrations of both drugs used for MM treatment in mice, are lower than those recommended in the current recommended dosage. Moreover, we showed that the VPA/PIO co-treatment increases the acetylation state of H3, compared to VPA treatment alone. Last, in addition to VPA, we investigated the effect of other HDACi, such as Vorinostat also known as suberanilohydroxamic acid (SAHA) or Mocetinostat (MGCD0103), in combination with PIO and we provided insight into the molecular mechanisms by which such combinatory therapy improves the anti-tumor effect of HDACi in MM.

## Materials and Methods

### Cell line

Human multiple myeloma cell line MOLP8 (DSMZ, Germany) isolated from a stage III MM Japanese patient in 2004 and was grown in RPMI-1640 medium (Lonza, Belgium) supplemented with 10% heat-deactivated fetal bovine serum (FBS) (Lonza, Belgium) at a density of 4x10^5^ cells/mL. Cells were maintained at 5% CO_2_ at 37°C and exponentially growing cells were used in all experiments.

### Animal Model

The establishment of MM mouse model was performed according to the Belgian regulation published in Royal Decree of 2010 giving the enforcement of protection and welfare of animals *‘Arrêté Royal du 6 avril 2010 relatif à la protection des animaux d’expérience’*. The protocol used was approved by the Local Ethical Commission of CER Group (Centre d’Economie Rural), Marloie Belgium, ahead of the initiation of the study.

10-week-old, female SOPF NSG mice (NOD.Cg-Prkdcscid Il2rgtm1Wjl/SzJ; #005557; Jackson Laboratories) were supplied by Charles River Laboratories, France. This animal model is B- and T-cell deficient with inactive NK cells (Natural Killer cells). Without previous irradiation, mice were injected intravenously (IV) in the tail vein with 3x106 MOLP8 cells (in 100μL of PBS), on day 0, to induce multiple myeloma. In order to assess the appearance and evolution of MM symptoms, mice were weighed every two days and underwent a general check-up such as grooming, eating frequency, general behavior and paralysis stage. Animals presenting a complete hind limb paralysis and a weight loss over 20%, were euthanized by manual cervical dislocation complying with the ethical regulations of the animal facility.

### Histochemistry H&E Staining & Radiography

A complete post mortem examination was performed on all mice and all abnormalities registered. Spleen, liver, lung and femurs/tibias were immediately fixed in 4% formaldehyde solution, buffered at pH6.9 (Merck Chemicals Ltd, Belgium) and processed in automated Shandon Citadel 1000 (Thermo, Belgium) for paraffin embedding, with previous decalcification for femurs and tibias. All samples were sectioned in 4μm sections with Shandon Finesse Microtome (Thermo, Belgium) and Hematoxylin and Eosin (H&E, both Sigma-Aldrich, Germany) staining was performed on tissue sections with Shandon Varistain Gemini Slide Stainer (Thermo, Belgium). Pictures were analyzed and captured with CellaVision DM96 automated microscope. Blind analysis was carried out by a clinical pathologist. In addition, the skeletons were analyzed by radiography to assess lytic lesions. X-rays were performed in the Centre Hospitalier Luxembourg (CHL) radiology department, on the breast x-ray scanner Microdose Spectra (Philips, Germany).

From all femurs, tibias, humerii and radii (except the ones fixed in formalin), bone marrow was flushed out. Cells were collected in RPMI1640 medium and filtered through 100μm filters (ImmunoTools, Germany), washed and centrifuged at 200g to get single cell suspensions. Bone marrow cells were used for protein, flow cytometry analysis and drug-resistance testing.

### Kappa/Lambda (κ/λ) Free Light Chain (FLC) quantification

Blood was taken from tail vein and collected into Microtainer K2E Tubes (Becton, Dickinson, The Netherlands), spun at 2000g for 10 minutes and plasma was assessed by FLC test, in the clinical diagnostic hematology lab of CHL. Freelite Human Kappa Free and Freelite Human Lambda Free kits (LK16 CB and LK018 CB; Roche, France) were used and measured on Cobas c501 reader (Roche, France).

### Treatment and Drugs

The drugs used, Valproic acid (VPA; Depakine IV, Sanofi Aventis, Belgium) and Pioglitazone (PIO; Actos, Takeda, Japan), are commercially available. VPA was dissolved in sodium chloride (0.9%) as a 2M stock solution and a pill of PIO was reduced to powder and dissolved in DMSO (AppliChem, U.S.A.), at a concentration of 20mM. DMSO was tested alone for side effects.

After establishment of the MM mouse model, we studied the effect of single agents or a combination of them (VPA, PIO and VPA/PIO). Mice were divided in different groups and treated by intraperitoneal injection (IP) on a daily basis, for 2 weeks. Starting at day 10, mice were injected either with phosphate buffered saline pH 7.4, (PBS, Gibco, Life Technologies, UK), VPA and PIO or with combination of the last two.

Mocetinostat (MGCD0103, MethylGene Inc., Canada) and Vorinostat (Suberoylanilide Hydroxamic Acid; SAHA; Selleck Chemicals, U.S.A.), have been also used in-vitro, in combination to PIO. 2×10^5^ MOLP8 cells were plated in culture flasks (Greiner, Belgium) and treated with MGCD0103 at 0.2μM, SAHA at 0.25μM or PIO at 30μM and combination of MGCD0103 and SAHA to PIO for 48 hours. BADGE (Bisphenol A Diglycidyl Ether, Sigma, Germany) has been used for its antagonist properties on PPARγ at 0.5μM concentration in co-treatment with PIO and HDACi.

### siRNA transfection

siPPARγ was purchased from Life Technologies, Belgium, antisense UAACGAAUGGGAUUUGUCtg and sense GACAAAUCACCAUUCGUUAtt and siGFP from Qiagen, Germany. MOLP8 cell line was transfected by using the protocol of Neon transfection system (Life Technologies, U.S.). Briefly, 60x10^4^ cells were re-suspended in 10μL of T solution and mixed with the appropriate concentration of siRNA. Cells were pipetted with the electroporation tips and pulsed with the Neon transfection system platform. Cells were transferred in 48 well-plate filled with 150μL of pre-warmed Complete medium (RPMI enriched with 10% of FBS).

### Flow cytometry

Extracellular staining was carried out in BM cells and on blood. Anti-mouse CD45 Fluorescein isothiocyanate (FITC) (IBL-5/25; Immunotools, Germany), GR1 Phycoerythrin (PE) (RB6-8C5; Immunotools, Germany), anti-human/anti-mouse CD44 Phycoerythrin-Cyanine7 (PE-Cy7) (IM7; eBioscience, Austria) were used to determine mice cells and discriminate them from the MM cell line stained with anti-human CD45 Allophycocyanin-Cyanine7 (APC-Cy7) (2D1), CD38 Peridinin chlorophyll protein-Cyanine 5.5 (PerCP-Cy5.5) (HIT2) and CD138 Allophycocyanin (APC) (MI15), all three provided by BD Bioscience, Netherlands. Data was acquired with BD FACS Canto flow cytometer and analyzed with BD FACS Diva software (BD Bioscience).

### Protein immunoblotting

Bone marrow cells were lysed, on ice, with a RIPA lysing buffer (Merck Millpore, U.S.), supplemented with protease inhibitors (Roche, Belgium), Phosphatase Cocktails 1 and 2 (Sigma-Aldrich, Germany), 10mM butyric acid (Aldrich, Germany) and Phenyl-methyl-sulphonyl-fluoride (PMSF; MP Biomedical, France). Protein concentrations were determined by Bradford assay (BioRad, U.S.).

For immunoblotting of anti-acetylated H3 rabbit polyclonal antibody (R&D Systems, U.K.) and anti-β actin mouse monoclonal antibody AC-15 (Sigma-Aldrich, Belgium), 40μg of protein samples were loaded and separated on 14% SDS-polyacrylamide gels. For immunoblotting of anti-caspase 3, rabbit polyclonal antibody and anti-PARP rabbit polyclonal antibody (both from Cell signaling, Netherlands), 20μg of protein were loaded and separated by 8% SDS-polyacrylamide gels. Proteins were transferred onto 0.2μm polyvinylidene difluoride (PVDF) membrane, blocked with 2% Enhanced chemiluminescent (ECL) Advance Blocking Agent (GE Healthcare, U.K.) and incubated overnight at 4°C with the primary antibodies. Goat-anti-rabbit and goat anti-mouse secondary antibodies (Jackson Immuno Research Laboratories, Inc., U.S.) were used adequately and detected with the ECL Advance detection kit (GE Healthcare, U.K.) on Kodak films (Thermo Fisher, Belgium).

Similarly, MOLP8 cells that have been treated with MGCD, SAHA and PIO, were harvested, lysed and protein concentration was determined as above. Immunoblotting was followed as described before.

### Cell viability (XTT) assay

4x10^4^ BM cells were incubated for 24hours in 96 well flat-bottomed cell culture plates with 100μL RPMI culture medium. Cells were then treated with VPA alone or in combination with PIO and incubated for further 48hours. Cell viability was determined by addition of 50μL of 1mg/mL XTT (Sigma) formazan solution, incubated for 3hours at 37°C, and the resulting color produced by viable cells, was measured with OPTIMA Elisa plate reader (BM6; LabTech) at 450nm and 630nm as a reference wavelength.

### Statistical analysis

The statistical significance of the treatments efficiency, between VPA or VPA/PIO and controls, was determined using Wilcoxon sum-rank test (p≤0.05), using Matlab (MathWorks Inc.). Survival rate between treatments versus control, is displayed in Kaplan-Meier plots.

## Results

### Development of the MM animal model

We developed an MM animal model displaying same symptoms as human MM, in order to extend our previous study in-vitro [21] and validate in-vivo the synergistic effect of the VPA/PIO combination. The MOLP8 cell line has been chosen based on our previous results showing that this cell line displayed synergistic index of the VPA/PIO, similar to that observed on isolated MM patient cells.

#### Follow-up of weight loss and MM development in NSG mouse model

**Fig 1:**
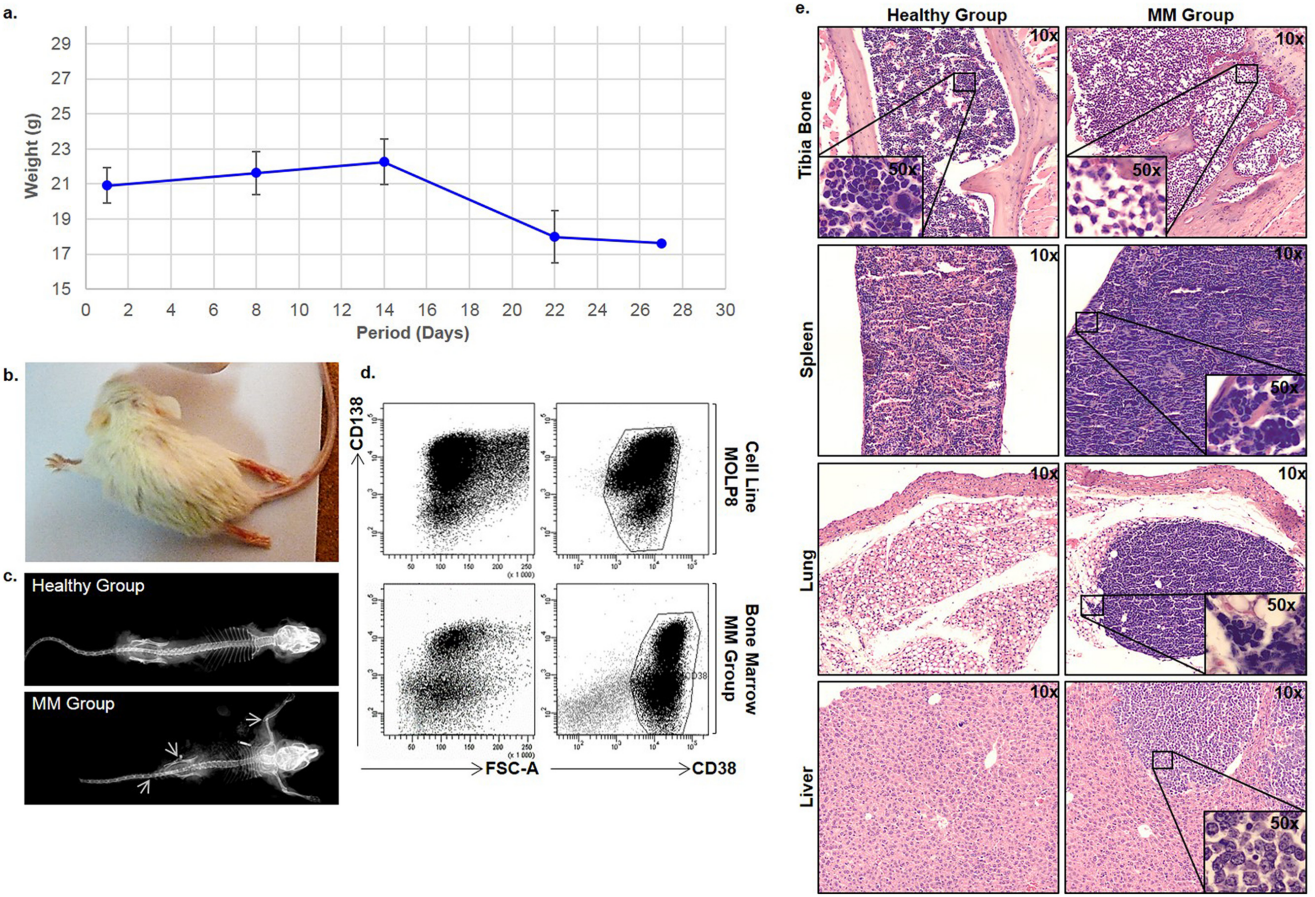
Development of MM mouse model. **a.** correlation between MM development and loss of weight in MM mice. Weight of mice inoculated with MOLP8 cell line reported as a median of the 6 mice and correlated the disease development. **b.** Paralysis development of back limbs, **c.** x-ray analysis of skeletons, showed osteolytic lesions in MM Group compared to healthy mice. **d.** Flow cytometry analysis of bone marrow-derived MOLP8 tumor cells. Cells, cultured in-vitro, are used as a control. **e.** H&E staining performed on longitudinal tibia sections showing a different cellularity and bone structure in the MM Group compared to healthy mice (Healthy Group). Also, H&E staining performed on spleen, lungs and liver of Healthy Group, is showing infiltration of malignant plasma cells in the MM Group. Internal panels are showing higher magnification (50x) for the identification of malignant cells infiltration.

In order to establish the animal model, we intravenously injected 3x10^6^ MOLP8 cells to NSG mice and monitored daily their clinical status and bi-daily basis the weight of animals. The clinical stages were determined by a veterinarian according to the following criteria: hind limp paralysis, ruffled hair coat, reluctance to move, hunched upper posture, slight segmentation of the lower vertebra and occasionally distended abdomen [22]. At day 18 after MOLP8 inoculation, a significant decrease of weight was observed without any other physical symptoms (Fig 1a). Therefore, our data establish a correlation between weight loss and disease development. Less than one week later, weight loss was followed by hind limp paralysis, ruffled hair coat, reluctance to move and eat as well as hunched posture (Fig 1b). As these symptoms were increased rapidly, the majority of animals were euthanized.

#### Osteolytic lesions detection

Using radiography, osteolytic lesions were detected in MM animals’ skeletons, compared to healthy mice. Dark areas indicated by arrows were observed in the spinal column and near the epiphysis of long bones, as well as in the pelvic bones, indicating the development of the disease (Fig 1c).

#### Flow cytometry analysis of bone marrow invasion by malignant cells

The invasion of malignant cells in bone marrow was assessed by analyzing cells expressing human plasma cell markers CD138+ and CD38+ [23] by flow cytometry using CD138+CD38+ MOLP8 cell line in culture as control. Our results showed that the bone marrow of mice injected with MOLP8 displayed CD138+CD38+ cell population, indicating a total invasion of malignant cells in their BM (Fig 1d).

#### Histological analysis of multiple myeloma mouse tissues

H&E staining on the BM of long bones sections of the MM Group, showed complete invasion of tumor cells compared to the Healthy Group, where normal cellularity, including granulocytes, erythroblasts, megakaryocytes and erythrocytes could be clearly observed. In addition, invasion of malignant plasma cells was also observed in lungs, liver and spleen but not in kidneys (Fig 1e).

### Effect of treatments

#### Combination of VPA-PIO decreases the invasion of cancer cells in bone marrow compared to VPA treatment alone

**Fig 2:**
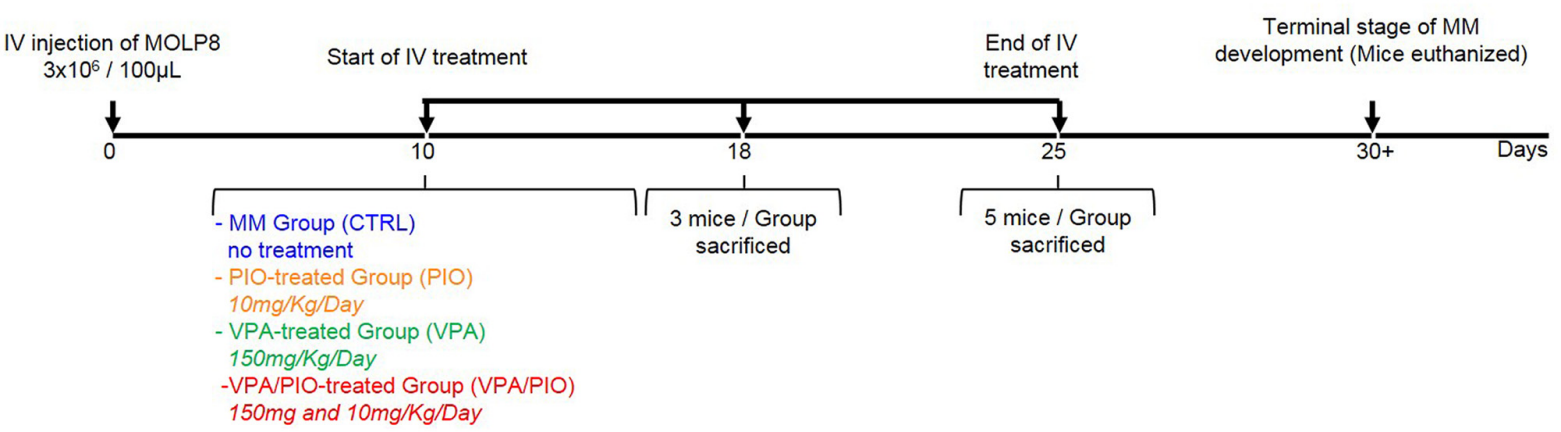
Experimental design. Experimental design of the treatment protocol of MM mouse model.

**Fig 3:**
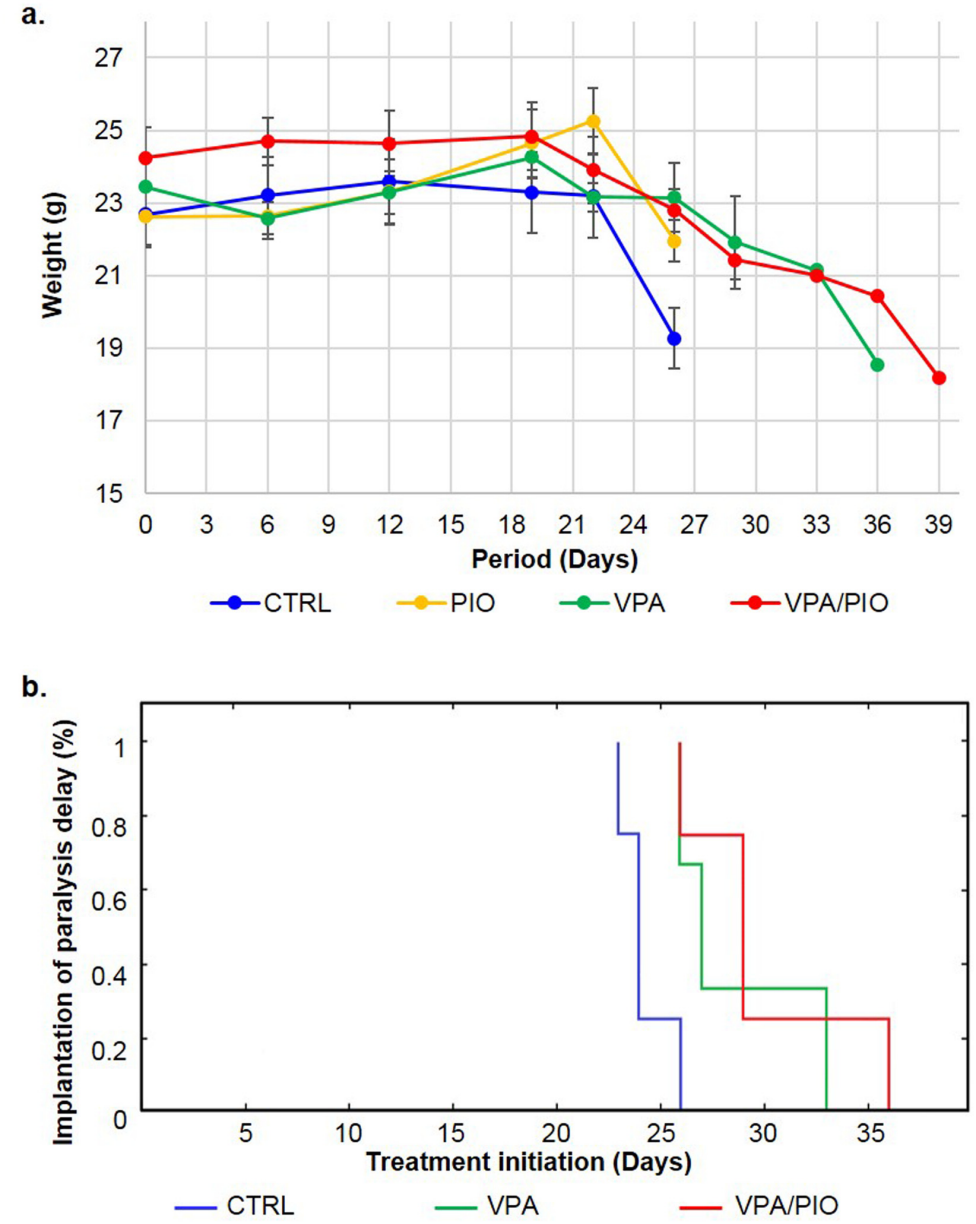
Effect of treatment on weight evolution in different MM mice groups. **a.** Effect observed of treatment on weight evolution in different MM mice groups and **b.** Kaplan-Meier curves sustaining the effect of treatments on the paralysis delay. Implantation to paralysis delay between MM Group (blue), VPA Group (green) and VPA/PIO co-treatment Group (red).

Based on our previous report, showing a synergistic effect of VPA and PIO combination on MM cell lines in-vitro, we studied the effect of these drugs, on our MM mouse model. As we first observed a weight loss before any other symptom, we decided to sacrifice the animals at various time points, in order to follow the efficiency of the treatment during disease development (Fig 2). Between day 21 and 24, CTRL (group without treatment) lost more than 15% of their weight, while animals under VPA/PIO or VPA treatments remained healthy. At day 25, all untreated animals of CTRL developed severe paralysis, exhibited high morbidity rate, whereas animals under VPA and PIO (VPA/PIO Group) and VPA (VPA Group) treatments showed the first signs of weight loss without developing any other signs of the disease. Although animals co-treated with VPA/PIO developed moderate weight loss, they exhibit prolonged survival compared to those received VPA alone (Fig 3a). Paralysis was significantly appeared earlier in un-treated group versus the co-treated animals (p≤0.03), as well as in the VPA treated animals (p≤0.04) (Fig 3b).

#### Measurement of lambda light chain in MM mice plasma treated with VPA or VPA/PIO

At day 18, the FLC assessment showed that the level of λ light chain secreted by MOLP8 cells in untreated mice was higher compared to that in VPA/PIO co-treated or VPA treated mice. More importantly, co-treated mice exhibited a level of λ light chain in plasma twice lower than those treated VPA alone (Table 1).

**Table 1:** FLC quantification in the different groups of mice at day18.

Animal Group	Treatment	λ light chain
**CTRL Group**	PBS	21.26 ± 2.4
**PIO Group**	PIO	19.7 (n/s)^b^
**VPA Group**	VPA	12.42 ± 1.02
**VPA/PIO Group**	VPA/PIO	5.8 ± 0.2

Average ± standard deviation (SD, n = 3)

^b^ non-significant (n/s, n = 1), the concentration of the λ light chain is in mg/mL.

#### Combination of VPA/PIO treatment improves the therapeutic benefit by decreasing the percentage of cancer cell invasion in bone marrow

**Fig 4:**
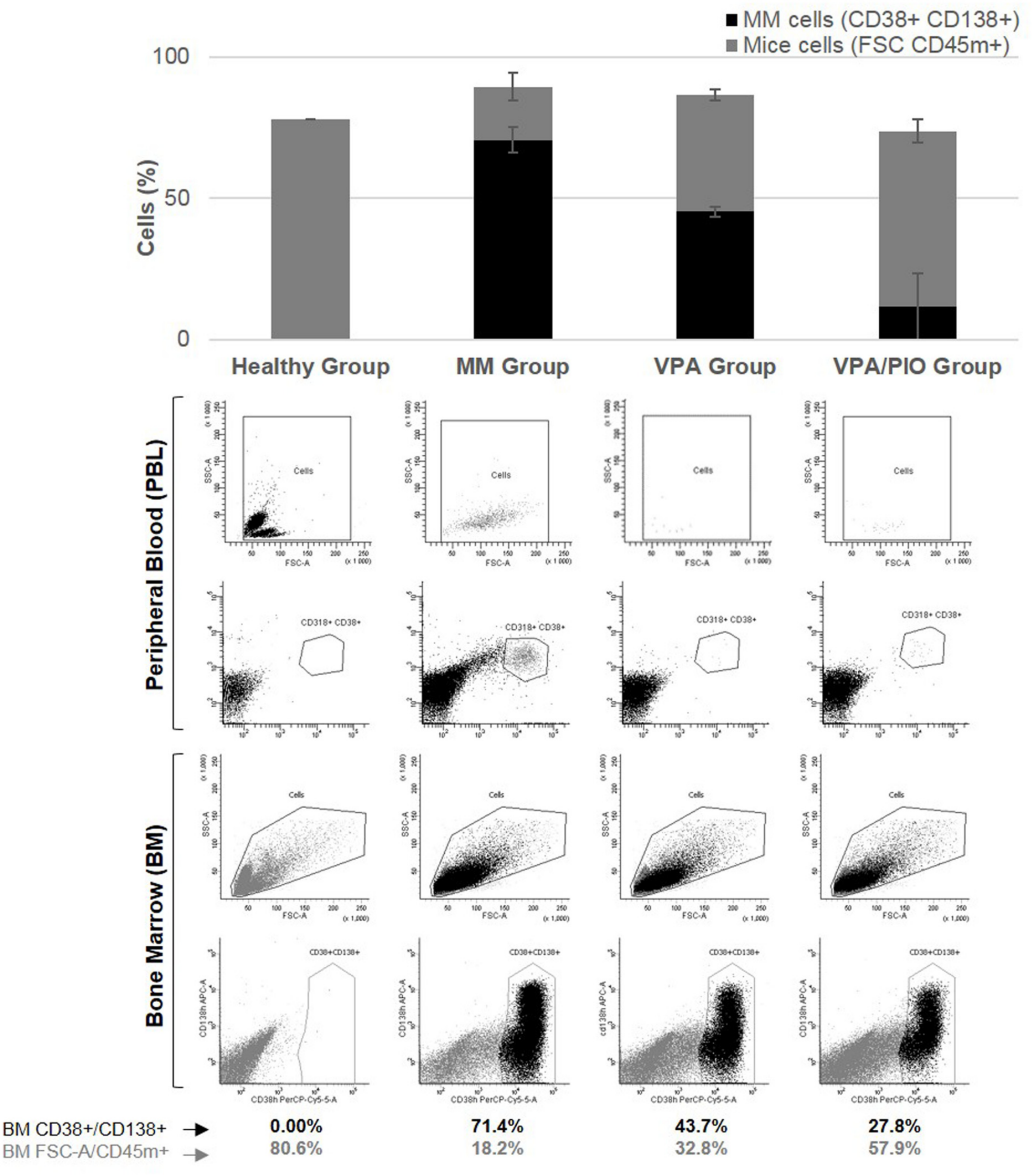
Flow cytometry analysis. CD38+CD138+ MOLP8 cell invasion in BM, as well as peripheral blood, was observed and compared between the different animal groups. Histogram representation shows the average of tumor burden for each group.

**Fig 5:**
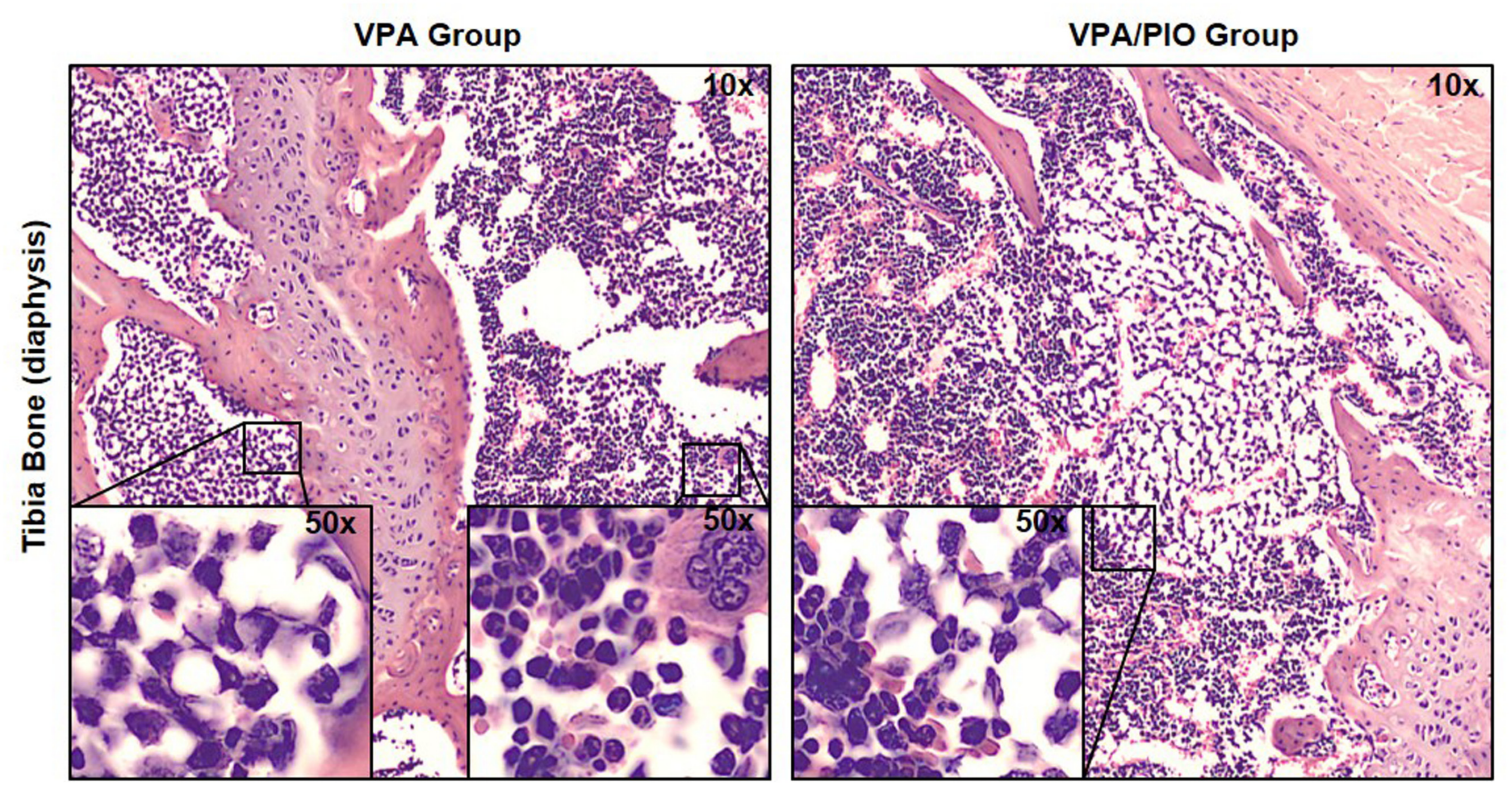
H&E staining of tibia sections. Blind reading of H&E staining revealed a difference in the level of infiltration of MM malignant cell plasma cells in treated animal groups, with either VPA/PIO or VPA alone. Internal panels are showing higher magnification (50x) for the identification of malignant cells infiltration.

To determine the efficacy of VPA/PIO in-vivo, we analyzed the percentage of MOLP8 cancer cells in the blood and bone marrow of mice. No cross reactivity between CD45m and CD45h was observed between the two species. As previously reported in Fig 1d, MOLP8 cells in-vitro are 100% CD138+CD38+. Our data showed no CD138+CD38+ plasma cells present in blood or bone marrow of healthy animals, however mice injected with MOLP8 cells showed 71.4% of CD138+CD38+ plasma cells in BM, while mice treated with VPA alone displayed 43.7% of CD138+CD38+ plasma cells in the BM, whereas PIO treatment alone had no effect. Interestingly, co-treatment of mice with VPA/PIO dramatically reduces the number of CD138+CD38+ plasma cells to 27.8%. The percentage of plasma cells in peripheral blood was lower than in the BM, but followed the same trend (Fig 4). H&E staining confirmed the absence of MM and the normal cellular characteristic of the bone marrow in CTRL Group, whereas a total invasion of tumor cells was observed in un-treated mice injected with MOLP8 cells. BM of mice treated with VPA/PIO or VPA alone showed partial invasion of the wall of the BM cavity by malignant cells, leaving the rest of the BM unaffected (Fig 5).

#### Combination of VPA/PIO increases acetylation level and induces apoptosis in multiple myeloma cells isolated from mice bone marrow

**Fig 6:**
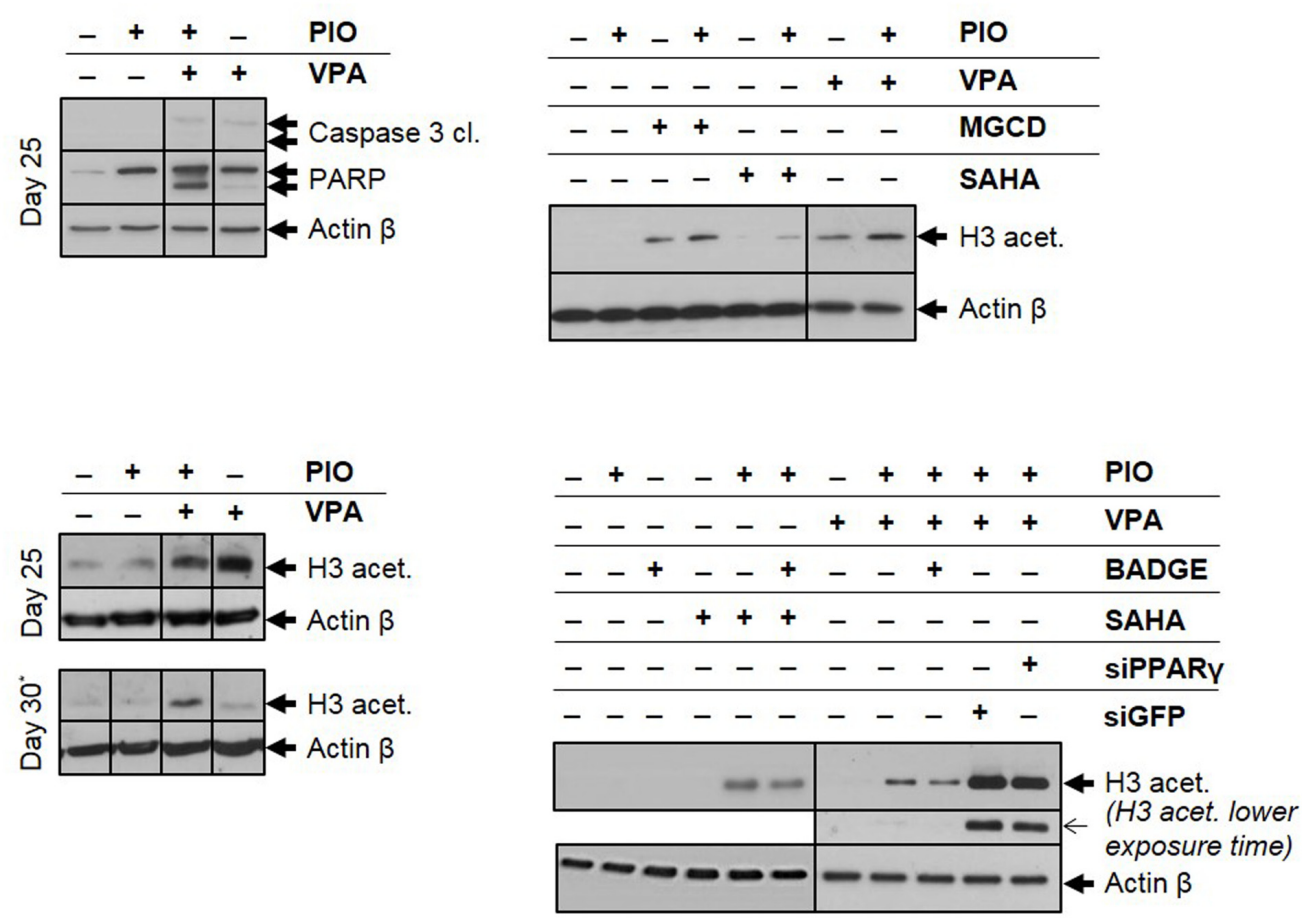
Analysis of cell death mechanism and H3 acetylation level. **a.** Immunoblotting of caspase 3 and PARP cleavages, **b.** H3 acetylation at day 25 and 30 in untreated (-) or treated (+) animals with VPA or/and PIO as indicated. *As negative control of day 30 was used the BM flush of the last survived mouse of the group, *with fully developed MM disease that was euthanized at day 25*. **c.** Effect of the combination of PIO with other HDACi on the H3 acetylation. MOLP8 cell line, treated with each of the three different HDACi (MGCD 0.2μM, SAHA 0.25μM and VPA 1mM) alone or combined with PIO 30μM. **d.** Study of the level of H3 acetylated of MOLP8 cells after treatment with the combination HDAC inhibitors/PIO and with the combination HDAC inhibitors/ PIO /PPARγ antagonist. Partial inhibition of H3 acetylation can be observed, by the pharmacological PPARγ antagonist, BADGE (0.5μM), or by genetic approaches using siGFP (as positive control) and siPPARγ after 48 hours of treatment.

We, next, investigated the molecular mechanism, underlying the effect of the combined therapy on the cell death of malignant plasma cells, isolated from mice treated with either VPA or PIO alone or in combination at days 25 and 30. We showed that malignant plasma cells from VPA/PIO Group displayed a higher cleavage of caspase 3 and PARP proteins, compared to those derived from VPA or PIO Groups, indicating that the combination of VPA/PIO is more effective at inducing tumor cell death compared to each treatment alone. Interestingly, the cells that displayed high caspase 3 and PARP cleavage, exhibited also a significant increase of H3 acetylation (Fig 6a). While at day 30, the VPA Group showed significant decrease of H3 acetylation, the level of H3 acetylated of the VPA/PIO Group was maintained, clearly suggesting that the VPA/PIO co-treatment sustains the acetylation of H3 compared to the VPA alone. The mechanism by which the acetylation of H3 is maintained, after the end of the VPA/PIO treatment, is currently under investigation (Fig 6b).

#### No development of drug resistance of cancer cells isolated from mice bone marrow treated with VPA or VPA/PIO

The comparative study of the cytotoxicity assay of VPA was carried out, using BM-derived MOLP8 cells and MOLP8 cell line cultured in-vitro. No significant difference was observed regarding the IC50, neither when used as single agent, either in combination with PIO (Table 2). Interestingly, at 1mM (IC20), which is the recommended dose of treatment [24], the percentage of cell survival was ~80% in VPA-treated MOLP8 cells in-vitro and in-vivo, while in cells treated with a combination of VPA/PIO under the same conditions, cell survival reached ~60% (Table 3).

**Table 2:** Cytotoxicity effect of VPA (IC50) on bone marrow isolated cells.

Sample		VPA IC50	
		VPA Single Agent	VPA Combination
**MOLP8 cells**		3.2 ± 0.0	1.7± 0.0
**Animal group**	**Control**	2.7 ± 0.3	1.9 ± 0.2
	**PIO**	3.2 ± 0.05	1.9 ± 0.2
	**VPA/PIO**	3.6 ± 0.6	2.15 ± 0.2
	**VPA**	3.4 ± 0.4	2.2 ± 0.5

Average ± standard deviation (SD, n = 3, triplicates).

IC50 is the concentration of VPA (mM) inducing 50 percent of cell death.

**Table 3:** Cell viability of bone marrow isolated cells at IC20 of VPA single or double treatment.

Sample		Cell Survival at IC20	
		VPA Single Agent	VPA Combination
**MOLP8 cells**		87 ± 4	58 ± 5
**Animal group**	**Control**	76 ± 7	57 ± 6
	**PIO**	80 ± 9	61 ± 5
	**VPA/PIO**	78 ± 4	61 ± 4
	**VPA**	81 ± 4	61 ± 5

Average ± standard deviation (SD, n = 4, triplicates).

Cell survival is the percentage of alive cells treated at the IC20 of drug

#### PIO increases the cytotoxicity and the acetylation properties of other HDAC inhibitors

To determine whether the therapeutic benefit of PIO is restricted to its association with VPA, we extended our study to other HDAC inhibitors such as Vorinostat (SAHA) and Mocetinostat (MGCD0103). The combination of PIO, with either SAHA or MGCD0103, increases both the cytotoxicity (Table 4) as well as the H3 acetylation in MOLP8 cells in-vitro when compared to the treatment of SAHA or MGCD0103 alone (Fig 6c). On the other hand, the treatment of cell line MOLP8 with PPARγ antagonist BADGE (Bisphenol A diglycidyl ether) in association with VPA/PIO diminish the cytotoxicity of the co-treatment VPA/PIO on MM cell line (Table 5) and the H3 acetylation state is also decreased. The inhibition of PPARγ expression with a siRNA followed with a co treatment VPA/PIO impede the elevation of H3 acetylated as it was observed before (Fig 6d).

**Table 4:** Cell viability of MOLP8 cells at the IC20 and IC50 of SAHA or MGCD0103.

Agent (cc.)	Cell survival at			
	IC20		IC50	
	Single Agent	PIO combination	Single Agent	PIO combination
**MGCD**	83.2± 3.3%	61.3± 3.5%	0.6± 0.04μM	0.37± 0.04μM
**SAHA**	80± 4.6%	60 ± 5%	0.55± 0.05μM	0.33± 0.05μM
**PIO**	91± 0%	-	-	-

Average ± standard deviation (SD, n = 4, triplicates).

The concentration of each drug used to determine the cell survival is 0.2 μM for MGCD, 0.25 μM for SAHA and 30μM for PIO.

**Table 5:** MOLP8 cell viability at IC20 of VPA after addition of PPARγ antagonist.

Agent (cc.)	Cell Survival at IC20		
	Single Agent	VPA/PIO	VPA/PIO + BADGE
**VPA**	76.7 ± 0.2%	64.07 ± 0.2%	71.7 ± 0.2%
**BADGE**	96.8 ± 0.8%	-	-
**PIO**	85.1 ± 0.2%	-	-

Average ± standard deviation (SD, n = 4, triplicates).

The concentration of each drug used to determine the cell survival is 1mM for VPA, 025 μM for BADGE and 30μM for PIO.

## Discussion

Despite major advances in the therapeutic approach of multiple myeloma, this disease remains incurable to date. In order to avoid or decrease the side effects of therapeutic combinations, like peripheral neuropathy [25], clinicians are always looking for new drugs [26] and new therapeutic associations [8]. Previously, we demonstrated that combining the HDACi, VPA with the PPARγ agonist, PIO increased the cytotoxic effect of the HDACi on MM cell lines, as well as on MM cells of patients [21]. To evaluate this combination in-vivo, we attempted to develop a MM mouse model that displays similar signs and symptoms as MM patients. Although, the major problem of MM xenograft models described so far is the premature death of animal due to rapid tumor progression, which makes the characterization difficult of such models [27]. In this study we followed the differential diagnosis of MM, appointed by the International Myeloma Working Group (IMWG) [28]. The three criteria that should be met in total are: presence of ≥10% clonal plasma cells in BM, presence of M-protein in serum or urine and end-organ damage, such as osteolytic lesions. These criteria differentiate MM clearly from solitary plasmacytoma (no clonal plasma cells in BM and normal skeletal survey). Using immunophenotyping, histology as well as bone x-rays methodologies, we showed that the bone marrow was completely invaded by malignant cells and that osteolysis, a hallmark of MM development [29].

The main challenge in drug development is to minimize toxicity. In clinical trials new drugs are tested at low concentrations [8] due to the unforeseeable side effects. In keeping with this, we have been choosing drugs already used in the clinic use for several years and evaluated their effect in mice using concentrations equal or below of those used in clinic. Thus, we tested the VPA/PIO combination at concentrations lower than those recommended for the treatment of epilepsy or type II diabetes respectively. Compared to other studies using VPA [30, 31], the concentration used here is significantly lower, (150mg/kg/day) [32]. Animals were treated with either PIO or VPA or in combination of both (Table 1). This study shows that the VPA/PIO combination treatment delays the MM development for more than one week in MM mice treated with the therapeutic combination compared to MM mice treated with VPA alone or to untreated mice. While the VPA treatment of MM mice extends their lifespan one week compared to untreated MM mice, the co-treatment VPA/PIO prolongs their lifespan for several days compared to the MM mice treated with VPA alone.

Further, we assessed the λ light chain fraction in the plasma using the FLC method, which is widely used as a prognostic and diagnostic marker to establish hematological diseases including MM in humans [33, 34]. Our results showed that the concentration of λ light chain is significantly higher in the CTRL Group than in the treated groups, indicating an advanced stage of the MM in the non-treated control group. In addition, the concentration of the λ light chain in the plasma of VPA/PIO treated mice is twice lower than in the plasma of VPA treated mice, indicating that the co-treatment is more efficient than the single agent VPA.

Another challenge of new drugs is to avoid drug resistance development which remains one of the main problems of cytotoxic cancer chemotherapy [25]. After two weeks of daily treatment, we have shown that tumor cells isolated from the bone marrow of both treated and untreated mice, display IC50 for VPA, similar to the cell line cultured in-vitro, thus providing an additional advantage of our model. One can therefore, conclude that the injected cells have not developed any drug resistance.

**Fig 7:**
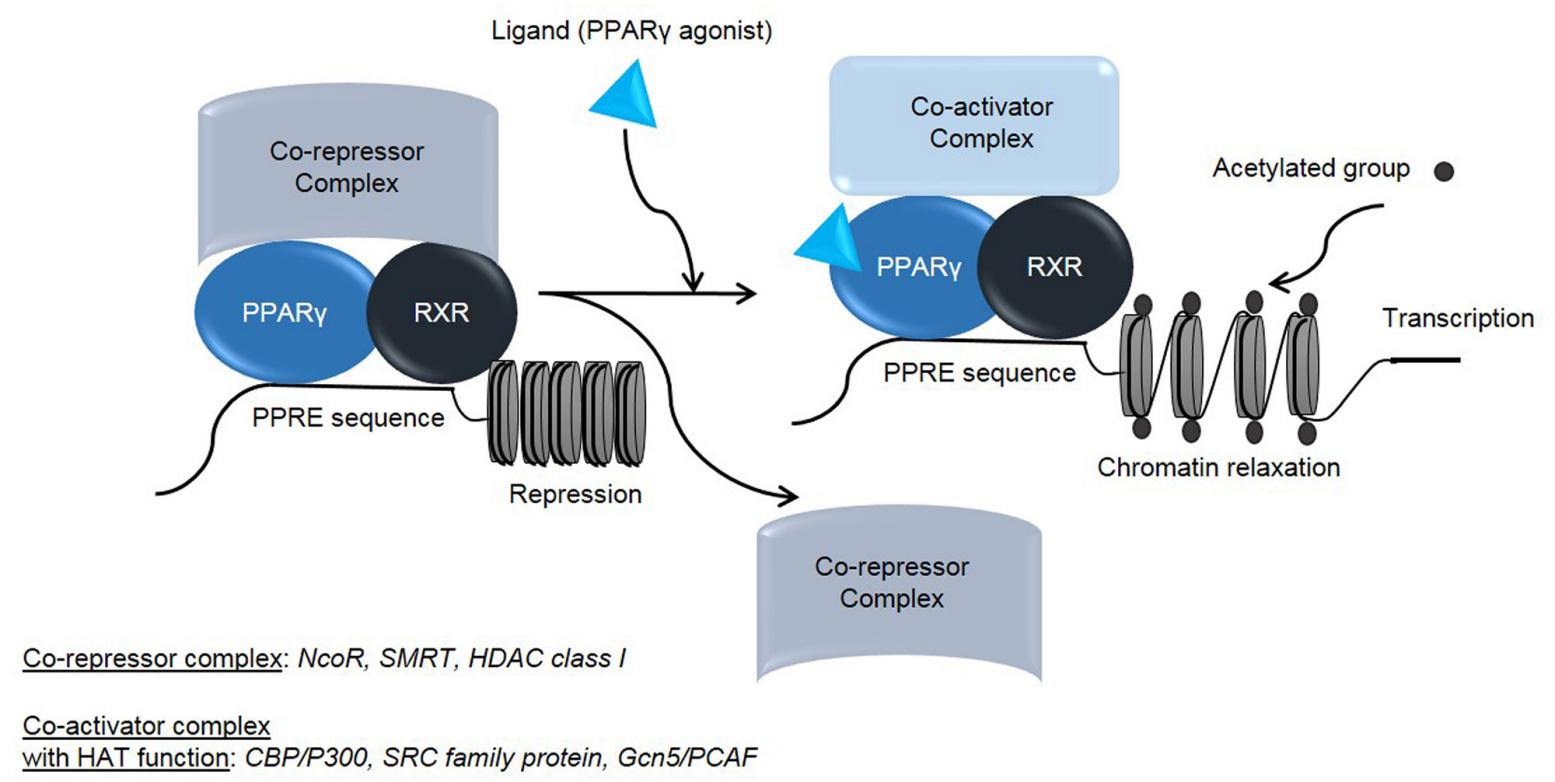
Molecular mechanism explaining the increase of acetylation level by PPARγ agonist. Schematic representation of molecular mechanism, explaining the increase of acetylation after PPARγ agonist treatment.

The anti-tumor activity of PPARγ agonists on tumor cell lines and animal models have been well described [19, 35, 36]. PPAR ligands are known to induce cell cycle arrest [37, 38] as well as differentiation and apoptosis in different cell line models [19, 35], however the molecular mechanism of their action as anticancer agents remains, so far, unclear. Although their effect on the lipid metabolism and glycemic control is well documented, thus, the PPARγ regulatory pathway has been extensively investigated. Briefly, PPARγ binds to RXR (Retinoid X Receptor) and forms a heterodimeric complex which interacts with cognate sequences in promoter regions of target genes, by binding to specific DNA sequence elements termed PPRE (Peroxisome Proliferator Response Element) [39]. In the absence of a ligand, PPARs/RXR heterodimer remains inactive through its binding with several co-repressors [40–42], while in the presence of a ligand, for either PPARγ or RXR, the co-repressors dissociate and the ligand can bind and activate several co-activators (Fig 7) [40]. In this regard, it has been reported that simultaneous treatments with ligands, specific for both PPARγ and RXR, has a synergistic effect on the transactivation of reporter genes. Several identified co-activators and co-repressors of PPARγ have intrinsic histone-modifying activities such as HATs and HDACs, which are acting as co-activators and co-repressors of PPARγ, respectively (Fig 7) [40, 43].

In this context, we believe that the mechanism of action of the VPA/PIO co-treatment on MM cells, could be similar to that described during the adipogenesis. Indeed, through its binding to the complex PPARγ/RXR, PIO induces the release of co-repressor complex and subsequently the association with a co-activator in order to increase the acetylation state of chromatin. Therefore, we speculate that the association of an HDAC inhibitor, such as VPA, synergizes the effect of PIO, by activating HATs, which results in higher chromatin expansion and prolongation of the acetylation state of H3, using low concentration of the PPARγ agonist (Fig 7).

Thus, in our study, PIO seems to act synergistically with VPA in order to increase the total state of acetylated H3, which has been illustrated by immunoblotting (Fig 6a and 6b), where can be observed increase of acetylated H3 compared to single agents VPA or PIO. The potentiation of PIO on VPA at low doses apply not only to VPA, but also to other HDACi, like MGCD0103 and SAHA used also at low concentrations (Table 4). Similarly to the combination of VPA/PIO, the co-treatment MGCD/PIO or SAHA/PIO in-vitro induces an increase of acetylated H3 compared to HDACi alone (Fig 6c).

Clinical trials supporting the use of HDACi in advanced MM patients reported, clinical benefit as well as tolerability when are treated with Bortezomib or Lenalidomide/HDACi regimens. So, it seems, that in combination therapies, HDACi could provide additional treatment options for the management of MM patients. Nevertheless, the aim of combining different drugs is to diversify the targeted pathways. Though, the mechanism by which the two drugs are acting on their targeted pathway is nevertheless different, as VPA inhibits class I and II HDACs and thus, it activates HATs, whereas PIO targets PPARγ. The two drugs synergize to induce a greater chromatin expansion and therefore a higher transcription level. In this study, it is important to highlight that this effect is achieved with a significantly lower drug concentrations, decreasing potential side effects.

## Conclusions

Currently, clinical trials are underway to study the effects of HDAC inhibitors in combination with other anti-neoplastic drugs in different types of hematological diseases. Therefore, it would be interesting to test VPA/PIO drug combination in a clinical study on MM patients, especially as the use of HDACi in MM clinical studies are pursued very actively [44].
